# Introducing Three-Dimensional Scanning for Phenotyping of Olive Fruits Based on an Extensive Germplasm Survey

**DOI:** 10.3390/plants11111501

**Published:** 2022-06-02

**Authors:** Ioanna Manolikaki, Chrysi Sergentani, Safiye Tul, Georgios Koubouris

**Affiliations:** Institute of Olive Tree, Subtropical Crops and Viticulture, ELGO DIMITRA, P.C 73134 Chania, Greece; manolikaki@elgo.iosv.gr (I.M.); sergentani@elgo.iosv.gr (C.S.); tulsafiye40@gmail.com (S.T.)

**Keywords:** breeding, image analysis, morphological characterization, olea europaea, plant phenotyping, phenomics

## Abstract

Morphological characterization of olive (*Olea europaea* L.) varieties to detect desirable traits has been based on the training of expert panels and implementation of laborious multiyear measurements with limitations in accuracy and throughput of measurements. The present study compares two- and three-dimensional imaging systems for phenotyping a large dataset of 50 olive varieties maintained in the National Germplasm Depository of Greece, employing this technology for the first time in olive fruit and endocarps. The olive varieties employed for the present study exhibited high phenotypic variation, particularly for the endocarp shadow area, which ranged from 0.17–3.34 cm^2^ as evaluated via 2D and 0.32–2.59 cm^2^ as determined by 3D scanning. We found significant positive correlations (*p* < 0.001) between the two methods for eight quantitative morphological traits using the Pearson correlation coefficient. The highest correlation between the two methods was detected for the endocarp length (r = 1) and width (r = 1) followed by the fruit length (r = 0.9865), mucro length (r = 0.9631), fruit shadow area (r = 0.9573), fruit width (r = 0.9480), nipple length (r = 0.9441), and endocarp area (r = 0.9184). The present study unraveled novel morphological indicators of olive fruits and endocarps such as volume, total area, up- and down-skin area, and center of gravity using 3D scanning. The highest volume and area regarding both endocarp and fruit were observed for ‘Gaidourelia’. This methodology could be integrated into existing olive breeding programs, especially when the speed of scanning increases. Another potential future application could be assessing olive fruit quality on the trees or in the processing facilities.

## 1. Introduction

The olive tree is regarded as one of the most symbolic tree species in the Mediterranean countries due to its cultivation for centuries. Some ancient olive trees are estimated to be over two thousand years old [1,2]. An expansion of olive growing outside the Mediterranean basin was realized during the past century. Recent statistical data depict the economic significance of olive growing with an estimated global average value of over USD 18 billion [3]. On top of that, the beneficial effects of consuming olive oil and table olives on human health have been widely documented [4,5].

Olive domestication was based on human observation of basic properties such as high fruit yield and large fruit size and tree adaptation to environmental stresses. Over time, it is estimated that more than 1500 varieties were accumulated worldwide, mainly through natural or human-driven selection and, to a lesser extent, through breeding [6]. During the 20th century, structured protocols were developed for the morphological description of olive varieties by the International Olive Council during the RESGEN project [7] and by the International Union for the Protection of New Varieties of Plants [8]. They included information regarding the tree, shoot, leaf, fruit, and endocarp traits allowing for the development of an ‘identity card’ of each variety and the publication of official plant variety catalogs [9].

This type of characterization is based on training expert panels and the implementation of laborious multiyear measurements [10], stressing the need for more technologically advanced and scientifically modern approaches for large-scale, fast, and precise plant behavioral studies. Fast and reliable methods combining molecular markers and advanced statistics are available [11]; however, the relevant authorities have not yet adopted them to catalog plant varieties. Therefore, phenomics might increase speed, reliability, and overall efficiency for morphological description and discrimination of large datasets of genetic resources.

The term phenome commonly refers to a compilation of traits, and the concurrent analysis of such a set of characters is referred to as phenomics [12]. Plant phenotyping is an essential means to tackle and comprehend plant–environment interaction and its interpretation into application in crop management practices [13]. Modern plant phenotyping relies on two fundamental aspects: (a) nondestructive measurements over time and (b) high-throughput measurements to compare a high number of genotypes [14]. Despite the rapid development of plant phenotyping during the past decades, significant advance is still needed to exploit new technologies and data management tools [15].

The agricultural sector in Greece faces serious challenges related to climate change vulnerability and limited investments in innovative solutions [16]. In addition, the adoption of new technologies for precision agriculture has met obstacles such as extra costs for investment in new equipment and trained employees [17]. Notably, a National Plant Phenotyping Network has not been established yet [16]. Few steps towards exploiting phenomics tools in the olive genetic resources of Greece have been recently reported [18,19,20].

Over the past decades, researchers have developed various imaging techniques for food quality determination and plant phenotyping for field crops using a wide range of methodologies [21]. Quick inspection, low cost, and consistent and accurate information via this technology are the most important advantages. For example, high-throughput imaging analysis has the potential to assess external fruit quality [22], such as color analysis of apple, citrus, mango, and banana [23,24,25,26], color, shape, and size of strawberry [27], size of apples [28], and shape of oranges [29]. ImageJ is one of the most widely known, open access, scientific image analysis software. Parameters such as the length, width, height, area, perimeter, and shape descriptors are extractable from ImageJ [30].

To date, three-dimensional (3D) imaging has been increasingly adopted for plant phenotyping as reassembly techniques improve and the cost of hardware decreases [31]. Being non-destructive, 3D scanning enables tracking of the architecture and development of plants through time [32]. Bernard et al. [33] presented an X-ray computed tomography method for the 3D characterization of walnut morphological traits. Recently, Rist et al. [34] established a fast and high-precision phenotyping pipeline for automated analysis of 3D point clouds and extraction of parameters related to grape bunch architecture. Plant phenotyping using 3D measuring devices may bridge the gap between plant function, agricultural traits, and genomics [35].

The present study compared 2D and 3D imaging systems for phenotyping a large dataset of olive varieties maintained in the National Germplasm Depository of Greece. The objectives were to (a) establish 3D point clouds of olive fruits and endocarps to assess phenotypic variation of the germplasm collection, (b) determine the correlation among 2D and 3D techniques for eight morphological traits, as well as (c) unravel novel morphological indicators of olive fruits and endocarps using 3D scanning.

## 2. Materials and Methods

### 2.1. Plant Material and Sample Preparation

Fruit and endocarp samples of fifty olive varieties (forty-six from Greece, three from Spain, and one from Italy) were considered for this study (Table 1). Fruit samples at the green-yellow and coloring stage according to the phenological color scale for the maturity of olive fruit [36] were manually collected in 2019 and 2020 from irrigated trees grown at the National Olive Germplasm Bank of Greece located in the Chrisopigi Monastery area (35°29′24″ N, 24°01′33″ E) near the Institute of Olive Tree, Subtropical Crops and Viticulture, ELGO-DIMITRA (Chania, Southern Greece). The mean air temperature for both years (2019 and 2020) in this area was 18.3 °C, relative humidity was 65.7%, and annual rainfall was 612 mm (H.A.O. meteorological station, Chania, Greece). Fruits were collected randomly, considering the fruit load, and avoiding extreme sizes of fruits. We stored all the samples in an environmentally controlled room at 7 °C until morphological evaluation during the same or the following day. After completing the morphological measurements on the fruit, we removed the pulp using a coarse fabric to isolate endocarps for subsequent analysis. Five hundred endocarps were soaked in 10% bleach for 5 min and stored in a dry place for morphological studies.

**Figure 1:**
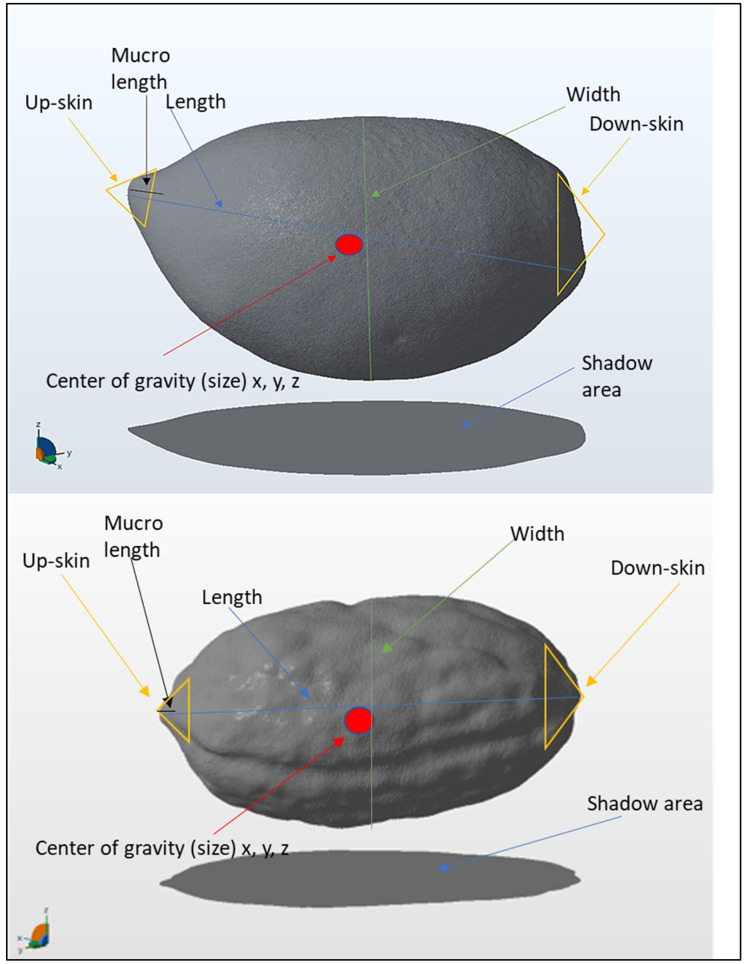
Morphological traits measured by 3D scanning and Autodesk Netfabb software analysis. All the morphological traits are explained in Table 2.

We measured the lengths, widths, shadow areas, volumes, total areas, up-skins, down-skins, centers of gravity (x, y, z), and nipple lengths of fruits; and the lengths, widths, shadow areas, volumes, total areas, up-skins, down-skins, centers of gravity (x, y, z), and mucro lengths of endocarps (Table 2 and Figure 1). Endocarp morphological traits are less influenced by the environmental conditions and farming systems than fruit, thus providing more accurate data for cultivar identification. The total samples of fruits and endocarps employed for 2D imaging across all varieties were 250 and 500, respectively; 5 fruit samples and 10 endocarp samples were analyzed for each variety. The same number of fruit and endocarp samples were 3D scanned as well.

### 2.2. Two-Dimensional Photography and Image J Analysis

For image capturing, a digital single-lens reflex (DSLR) camera (Nikon Coolpix B500, Nikon Inc., Tokyo, Japan) was placed on the top of a portable photo box facing the sample and fixed on a solid arm. The objects were placed inside a photo box illuminated with two white LED light sources with an illumination of 5500 k. We used a light-blue or black background depending on the sample type. We intended to mimic natural daylight illumination and avoid shadows. We set up the camera with a focal length of 59 mm (35 mm lens) so that the field of view was large enough to accommodate ten endocarp or five fruit samples of each cultivar. The distance between the lens and the sample was 40 cm allowing maximum visualization of the samples without occlusion of the mucro or nipple. The camera was adjusted in manual mode, with an aperture of f/4, an exposure time of 1/250 s, and an ISO speed rating value of 125. The captured images were acquired and saved in JPG format, with 4608 × 3456 pixels in resolution, a pixel density of 300 dpi, and a color depth of 24 bits.

**Figure 2:**
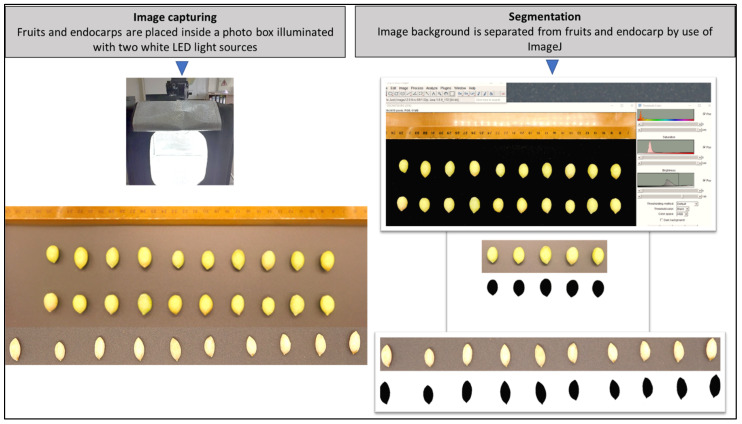
Workflow of olive endocarp and fruit 2D photography, acquisition of measurements, and ImageJ software analysis (ImageJ 1.52p).

Segmentation separates the image background from the object and provides a binary image from which we can derive a mathematical representation of shape. Regarding 2D image segmentation, we used ImageJ software (ImageJ 1.52p). We employed the color thresholding technique by modifying manually the values of ‘Saturation,’ ‘Brightness,’ and ‘Hue’ of each image. Figure 2 depicts the 2D image segmentation procedure.

### 2.3. Three-Dimensional Scanning and Autodesk Netfabb Software Analysis

**Figure 3:**
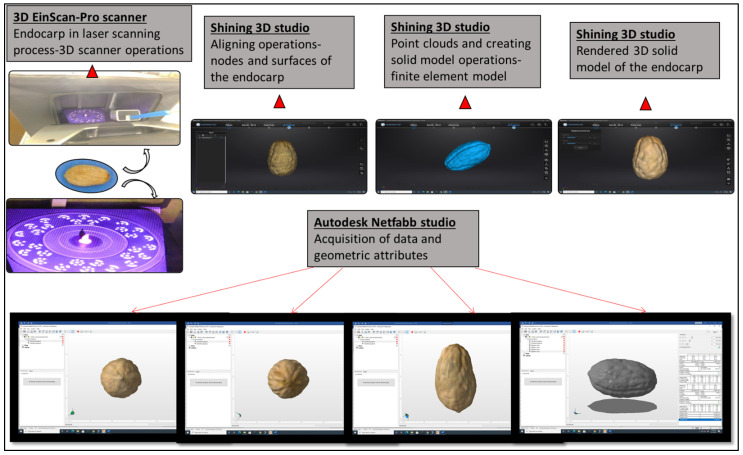
Workflow of olive endocarp three-dimensional scanning, acquisition of measurements, and Autodesk Netfabb software analysis.

In creating 3D data of olive fruit and endocarp samples, we utilized an Einscan-Pro 3D Desktop scanner (Shining 3D Tech Co. Ltd., Hangzhou, China). The sensor technology is based on blue LED structured light. The dense point clouds have a mesh resolution of up to 0.16 mm and point accuracy of up to 0.05 mm. We mounted Olive samples on a turntable. The scanning procedure comprised forty scanning steps for the whole sample surface in two-sided vertical and horizontal positions. We set up HD resolution at macro range options for the scanning process and the total scanning time of each sample was 20 min. Scanned surfaces were aligned and remeshed into the Shining 3D software. Shining 3D software was also used for ordering surface mesh structure and final surface refining of the solid model (Figure 3). We measured the morphological traits in the Autodesk Netfabb software (Autodesk^®^ Netfabb^®^ 2019.0© 2018 Autodesk, Inc., San Rafael, CA, USA), and some virtual outputs are shown in Figure 3.

### 2.4. Statistics

Statistical analysis was conducted using SPSS software (Version 22, SPSS Inc., Chicago, IL, USA). Descriptive statistics analysis and Pearson correlation coefficient were implemented in order to validate 3D morphological data with 2D data. One-way analysis of variance (ANOVA) was employed for data analysis of 3D morphological traits. The least significant difference (LSD) test at *p* = 0.05 was employed to detect statistically significant differences among cultivars.

## 3. Results

### 3.1. Assessing Phenotypic Variation of the Germplasm Collection from 2D Images and 3D Scanning

**Figure 4:**
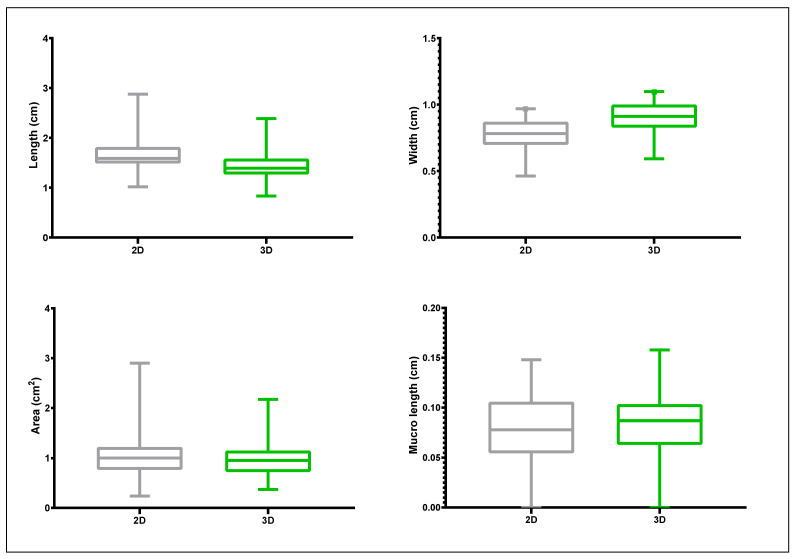
Box-plots of olive endocarp (500 replications) morphological traits measured by 2D photography and Image J analysis and 3D scanning and Autodesk Netfabb software analysis.

**Figure 5:**
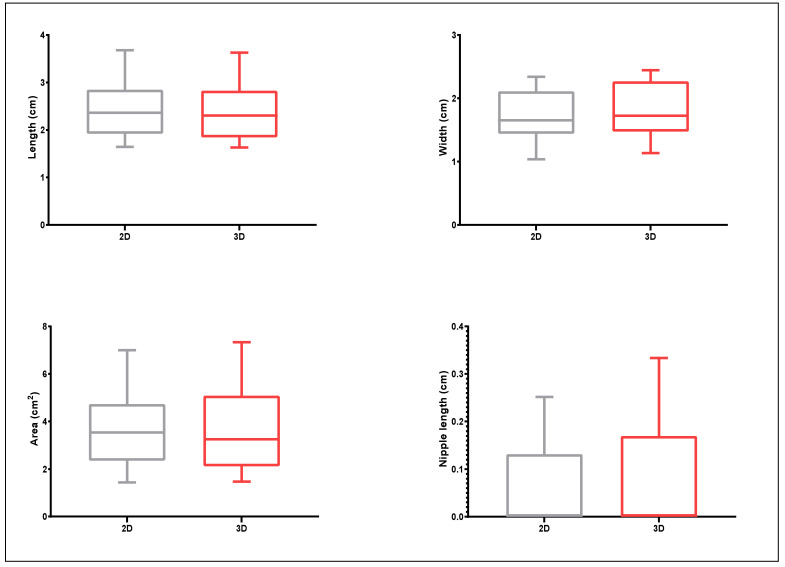
Box-plots of olive fruit (250 replications) morphological traits measured by 2D photography and Image J analysis and 3D scanning and Autodesk Netfabb software analysis.

An extensive Germplasm collection consisting of 50 olive genotypes was evaluated by 2D and 3D scanning for eight quantitative traits whose descriptive statistics (mean, standard deviation, minimum and maximum) are given in Figure 4 and Figure 5. The olive varieties employed for the present study exhibited high phenotypic variation in morphology-related traits, particularly for the endocarp shadow area, which ranged from approx. 0.17 cm^2^ for ‘Koroneiki’ to more than 3.34 cm^2^ for ‘Gaidourelia’ according to 2D and from 0.32 cm^2^ to 2.59 cm^2^ as determined by 3D scanning. Mucro length ranged from 0 cm for twelve cultivars to 0.24 cm for ‘Gaidourelia’ according to 2D and 0–0.25 cm according to 3D. Endocarp length ranged from 0.87 cm for ‘Arbequina’ to 3.38 cm for ‘Gaidourelia’ based on 2D and 0.75–3.26 cm according to 3D. Endocarp width ranged from 0.44 cm for ‘Koroneiki’ to 1.23 cm for ‘Petrolia’ when evaluated through 2D and from 0.57 to 1.36 cm in the case of 3D.

In the case of fruit samples, length ranged from 0.86 cm for ‘Chrisolia’ to 3.83 cm for ‘Amigdalolia’ via 2D and from 0.87–3.74 cm according to 3D. Fruit width ranged from 0.88 cm for ‘Chrisolia’ to 2.72 cm for ‘Kolimbada’ as analyzed by 2D and from 0.87 cm for ‘Chrisolia’ to 2.75 cm for ‘Karidolia’ by 3D processing. Fruit shadow area varied from 0.7 cm^2^ for ‘Chrisolia’ to 7.73 cm^2^ for ‘Karidolia’ based on 2D and from 0.6 cm^2^ for ‘Chrisolia’ to 7.82 cm^2^ for ‘Gaidourelia’ based on 3D. Fruit nipple length ranged from 0 cm for twenty-nine cultivars to 0.35 cm for ‘Amigdalolia’ assessed with 2D and 0 to 0.41 for 3D.

### 3.2. Proof-of-Principle on Olive Cultivars: Comparison of 2D and 3D Methods

**Figure 6:**
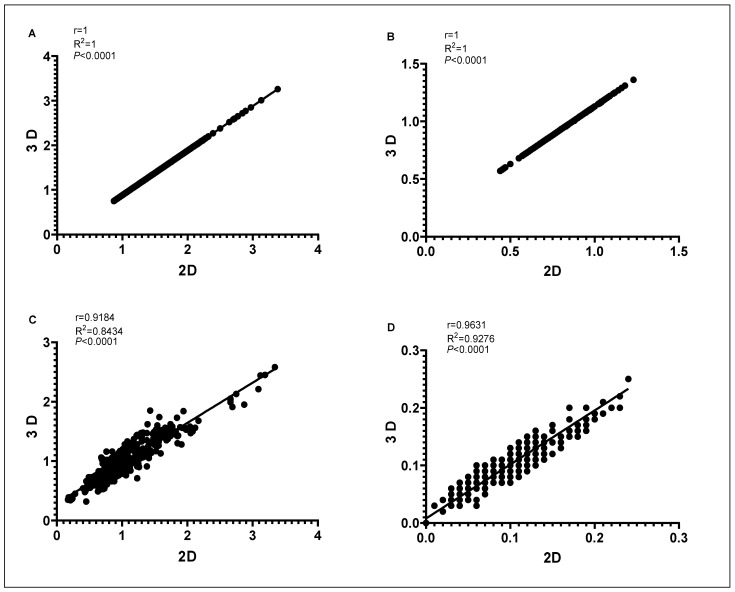
Correlation coefficient (r) with corresponding *p*-values of the extracted parameters for the olive endocarp length (**A**), width (**B**), shadow area (**C**), and mucro length (**D**) between 2D and 3D scanning.

**Figure 7:**
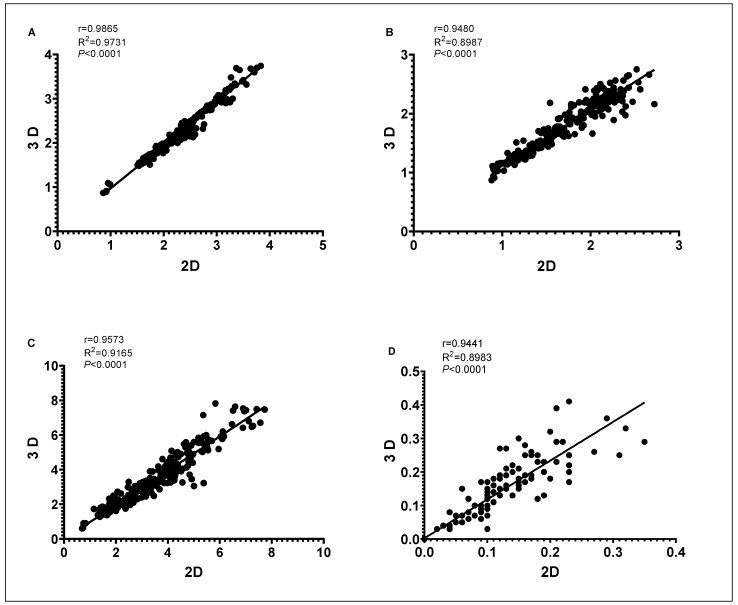
Correlation coefficient (r) with corresponding *p*-values of the extracted parameters for the olive fruit length (**A**), width (**B**), shadow area (**C**), and nipple length (**D**) between 2D and 3D scanning.

The outputs of fruit and endocarp morphological analysis through 2D and 3D methods were tested for correlation. Correlation plots of 8 morphological traits derived using the two methods are shown in Figure 6 and Figure 7.

Using Pearson correlation coefficient, we found significant positive correlations (*p* < 0.001) between the two methods for the 8 quantitative morphological traits. The highest correlation between the two methods was detected for endocarp length (r = 1) and width (r = 1) followed by fruit length (r = 0.9865), mucro length (r = 0.9631), fruit shadow area (r = 0.9573), fruit width (r = 0.9480), nipple length (r = 0.9441) and endocarp shadow area (r = 0.9184).

**Figure 8:**
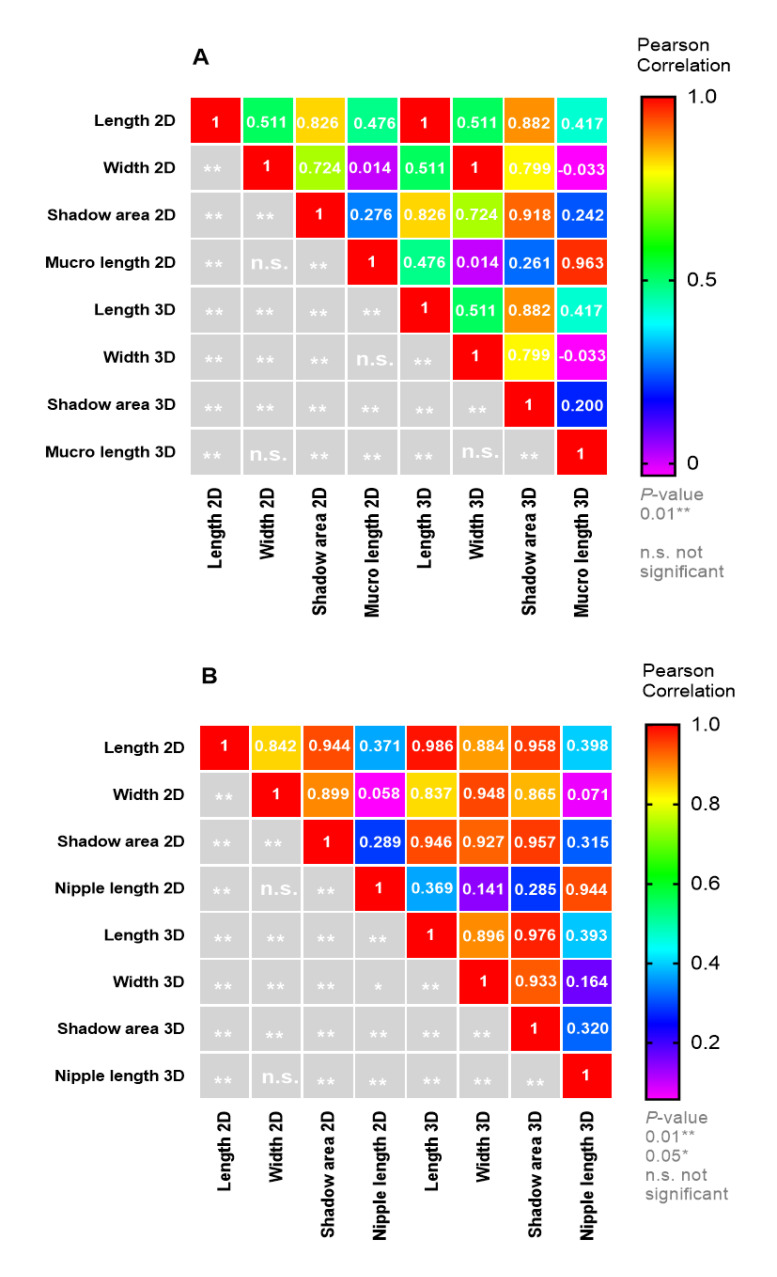
Pearson correlation matrix for olive (**A**) endocarp morphological traits, and (**B**) fruit morphological traits.

Moreover, using a correlation matrix, we found significant (*p* < 0.01) positive correlations between the eight common quantitative morphological traits presented in Figure 8. We observed that the endocarp length was positively correlated with the endocarp shadow area (0.826 and 0.882, for 2D and 3D, respectively). The endocarp width was positively correlated with the endocarp shadow area (0.724 and 0.799, for 2D and 3D, respectively) to a less extent than length, indicating that the endocarp shadow area depends on the length more than the width. We also observed moderate positive correlations between the endocarp length and the mucro length (0.476 and 0.417, for 2D and 3D, respectively). We found an even lower correlation between the endocarp shadow area and mucro length (0.276 and 0.200, for 2D and 3D, respectively). No correlation was recorded between the endocarp width and mucro length via 2D (0.014) and via 3D (−0.033).

In addition, data analysis for fruit traits showed a positive significant (*p* < 0.01) correlation coefficient of the fruit length with the fruit shadow area (0.944 and 0.976, for 2D and 3D, respectively). A lower positive significant (*p* < 0.01) correlation between the fruit width and the fruit shadow area (0.899 and 0.933, for 2D and 3D, respectively) was recorded. Furthermore, a moderate positive significant (*p* < 0.01) correlation between fruit length and fruit nipple length (0.371 and 0.393, for 2D and 3D, respectively) and a low positive significant (*p* < 0.01) correlation between the fruit shadow area and the fruit nipple length (0.289 and 0.320, for 2D and 3D, respectively), was observed. No correlation was detected between fruit width and nipple length (0.071 and 0.164, for 2D and 3D, respectively).

### 3.3. Evaluation of Phenotypic Variation of the Germplasm Collection Based on Traits Exclusively Derived from 3D Scanning

Three-dimensional methods can be exploited to identify the shape of fruits and endocarps by analyzing additional parameters which cannot be addressed by 2D images. High phenotypic variation was detected among the varieties for the traits exclusively derived from 3D scanning (Supplementary Tables S1 and S2). The highest endocarp volume and area were observed for ‘Gaidourelia’ (1.24 cm^3^ and 7.20 cm^2^), while the lowest for ‘Koroneki’ (0.12 cm^3^ and 1.28 cm^2^). Petrolia showed the highest up-skin area (0.36 cm^2^), while a range of varieties had the lowest (0 cm^2^). The lowest up-skin area was observed for the varieties ‘Asprolia Lefkados’, ‘Dopia Zakinthou,’ ‘Frantoio,’ ‘Galatistas,’ ‘Kalamon,’ ‘Karolia,’ ‘Kolireiki Ilias,’ ‘Koroneiki,’ ‘Koutsourelia,’ ‘Lefkolia Serron,’ ‘Lianolia Kerkiras,’ ‘Mastoidis,’ ‘Matolia Ilias,’ ‘Mavrelia,’ ‘Megaritiki,’ ‘Rachati,’ Thiaki,’ and ‘Throubolia.’ The highest down-skin area was detected in ‘Kolimbada’ (0.26 cm^2^), and the lowest (0 cm^2^) in ‘Karolia,’ ‘Kolireiki Ilias,’ ‘Koroneiki,’ ‘Koutsourelia,’ ‘Lianolia Kerkiras,’ ‘Manzanilla,’ ‘Mastoidis,’ and ‘Rachati.’

Concerning fruit traits, ‘Gaidourelia’ showed the highest volume (11.28 cm^3^) and area (25.98 cm^2^), while ‘Chrisolia’ had the lowest (0.48 cm^3^ and 3.01 cm^2^). The lowest up-skin and down-skin areas were observed for ‘Asprolia Lefkados’ (0.21 cm^2^ and 0.30 cm^2^), while ‘Amfissis’ and ‘Karidolia’ showed the highest up-skin (2.43 cm^2^) and down-skin (2.19 cm^2^) areas, respectively.

## 4. Discussion

The present study presented three-dimensional scanning of fruit and endocarps for a large dataset of olive varieties for the first time in the literature. Image analysis, especially 3D, has been previously applied in food science for phenotyping purposes [33,37], albeit olive fruit phenotyping via 3D analysis has not been considered yet. We observed sufficient concordance between 2D and 3D measurements of length, width, shadow area, mucro, and nipple length. Based on our results from the correlation among the eight traits, the longer the endocarp is, the larger the shadow area is, and the wider the endocarp is, the shorter the mucro is. Similar results were observed for the fruit size and nipple length. The dataset obtained from 2D and 3D image analysis included information about the fruit size that is of great interest to farmers and breeders. The three-dimensional analysis is a suitable method to select interesting genotypes for a breeding program to combine many favorable traits in a new variety. Thus, accessions with high length, width, and shadow area could be suitable for breeding new varieties destined to produce table olives.

The three-dimensional analysis presents numerous advantages compared to classical morphological evaluation, mainly the higher accuracy of measurements. Some errors that might be induced in the manual assessment are the axis of measurement determined by the eye, potentially resulting in non-maximal distances or non-orthogonal axes. Moreover, the 2D method has limitations imposed by the user, such as the separation of samples from the image background and the different sample positions for image acquisition. Of these two methods for determining the geometric parameters of olive accessions, 3D scanning is considered the most informative approach. The models generated using the 3D method support the determination of a full range of geometric parameters (area, volume, up-skin and down-skin area, center of gravity) of whole fruits and their fragments, impossible to measure via 2D.

Moreover, the shape of the analyzed fruit is stored in the computer memory as a cloud of points. It can be used for future research to measure all the geometric parameters without using displacement methods [38]. In addition, Rist et al. [34] presented a non-invasive and non-contact field application that facilitates high-precision phenotyping of grapevine bunch traits, such as the berry number, using a 3D scanner and automated analysis software. Furthermore, one of the most pronounced uses of 3D vision-based phenotyping techniques is the development of 3D models of whole plants with organ-level labeling.

Dutagaci et al. [39] provided a data set composed of 3D models acquired through X-ray scanning of rosebush plants which can be used for the evaluation of automatic phenotyping methods beyond classifying plant points as stems and leaves. The three-dimensional method is also efficient for fruit quality determination. Bernard et al. [33] reported that the characterization of walnuts using X-ray CT is suitable to phenotype the fruit quality and support correlations between the morphometric traits. The same authors suggested that in addition to selecting superior genotypes, deep learning methods can either develop an application for food security and detection of commercial frauds purposes or identify infection by pathogens. However, the time consumed by the user can constitute a limitation of its use. In the present study, two-dimensional acquisition included multiple samples, while the three-dimensional workflow included a single sample that took longer time to be acquired.

However, we elucidated novel morphological indicators of olive fruits and endocarps using 3D scanning that could not be acquired via 2D photographs. Other applications, such as structure from motion (SfM) which use a set of 2D images captured by RGB cameras to reconstruct a 3D model from the object of interest, focus on reproducibility but not on accuracy. SfM approaches need a short time for capturing the image but need much effort for the algorithm reconstruction [40]. Despite the fact that 3D models are time-consuming, further automation of the whole process or scanning several objects at once, could be an efficient improvement in reducing time, especially for consecutive changes of object position. The use of imaging systems carried by a robotic manipulator can provide a viable solution to this issue, due to its flexibility to position and orient cameras at the best-intended viewpoints [41].

## 5. Conclusions

The present study compared 2D and 3D imaging systems for phenotyping a large dataset of olive varieties maintained in the National Germplasm Depository of Greece. We established 3D point clouds of olive fruits and endocarps to assess phenotypic variation of the Greek Germplasm Collection. This database could be enriched in the network of the International Olive Council, which harbors over 1000 olive varieties in several countries. We also determined the correlation among 2D and 3D techniques for eight morphological traits verifying the high accuracy of the proposed method for morphological measurements required by international organizations such as the Community Plant Variety Office (CPVO) and the International Union for the Protection of New Varieties of Plants (UPOV) and so far, implemented through time-consuming, low-accuracy and non-replicable human observation. The present study unraveled novel morphological indicators of olive fruits and endocarps using 3D scanning that could not be acquired via 2D photographs. This methodology could be integrated into existing olive breeding programs, especially when the speed of scanning increases. Another potential future application could be assessing olive fruit quality on the trees or in the processing facilities.

## Figures and Tables

**Table 1:** List of fifty olive varieties used for the study.

Varieties				
Adramitini	Dafnelia	Kolireiki Ilias	Mastoidis	Pierias Skotiniotiki
Aggouromanakolia	Dopia Zakinthou	Kolimbada	Matolia Ilias	Pikrolia
Amfissis	Frantoio	Koroneiki	Mavrelia	Rachati
Arbequina	Frantoio Rodou	Kothreiki	Mavrelia Serron	Stroggilolia
Amigdalolia	Gaidourelia	Koutsourelia	Mavrelia Lefkadas	Thiaki
Asprolia $$$Alexandroupolis	Galatistas	Lefkolia Serron	Megaritiki	Throumbolia Thassou
Asprolia Lefkados	Kalamon	Lianolia Kerkiras	Mirtolia	Throubolia
Chalkidikis	Kalokerida	Lianomanako Tirou	Petrolia	Tragolia
Chondrolia Chalkidikis	Karolia	Makris	Picual	Valanolia
Chrisolia	Karidolia	Manzanilla	Pierias	Vasilikada

**Table 2:** Morphological traits measured by 2D photography and ImageJ analysis and 3D scanning and Autodesk Netfabb software analysis.

Morphological Trait	Unit	Method	
		2D	3D
Length	cm	The maximum length from the base to the end (major axis)	Greatest dimension orthogonal to the height
Width	cm	Widest point perpendicular to the major axis	Greatest dimension orthogonal to both height and length
Shadow area	cm^2^	The size of the region enclosed within it.	Shadow area
Mucro length	cm	The maximum length from the base to the end (major axis) of mucro	The maximum length from the base to the end (major axis) of mucro
Nipple length	cm	The maximum length from the base to the end (major axis) of nipple	The maximum length from the base to the end (major axis) of nipple
Volume	cm^3^	not applicable	A scalar quantity expressing the amount of three-dimensional space enclosed by a closed surface.
Area	cm^2^	not applicable	Total surface area, or the sum of the areas, on every outward surface
Up-skin (cm^2^)	cm^2^	not applicable	Upwards facing surface at a specific angle (45°)
Down-skin (cm^2^)	cm^2^	not applicable	Downwards facing surface at a specific angle (45°).
Center of gravity (size) x, y, z	cm	not applicable	The point where all the weight of the object can be considered to be concentrated. (x, y, z coordinate)

## Data Availability

Data are available by the authors upon reasonable request.

## Supplementary Materials

The following supporting information can be downloaded at: https://www.mdpi.com/article/10.3390/plants11111501/s1, Table S1: Endocarp 3D morphological traits of 50 olive varieties.; Table S2: Fruit 3D morphological traits of 50 olive varieties.

## References

[1] Rackham O., Moody J. (1996). The Making of the Cretan Landscape.

[2] Bombarely A., Doulis A.G., Lambrou K.K., Zioutis C., Margaritis E., Koubouris G. (2021). Elucidation of the Origin of the Monumental Olive Tree of Vouves in Crete, Greece. Plants.

[3] FAOSTAT (2018). Food and Agriculture Organization of the United Nations. Statistics Division..

[4] Diamantakos P., Ioannidis K., Papanikolaou C., Tsolakou A., Rigakou A., Melliou E., Magiatis P. (2021). A new definition of the Tterm “high-phenolic olive oil” based on large scale statistical data of greek olive oils analyzed by qNMR. Molecules.

[5] Grounta A., Tassou C., Panagou E., Shahidi F., Kiritsakis A. (2017). Greek-Style Table Olives and Their Functional Value. Olives and Olive Oil as Functional Foods: Bioactivity, Chemistry and Processing.

[6] Olea Databases National Research Council of Italy. http://www.oleadb.it/.

[7] Barranco D., Cimato A., Fiorino P., Rallo L., Touzani A., Castaneda C., Serafıni F., Trujillo I. (2000). World Catalogue of Olive Varieties.

[8] International Union for the Protection of New Varieties of Plants (UPOV) (2002). Technical Guideline for the Conduct of Tests for Distinctness, Homogeneity, and Stability in Olive.

[9] European Commission (EC) Plant Variety Catalogues, Databases & Information Systems. Commission Implementing Directive 2014/97/EU..

[10] Koubouris G.C., Avramidou E.V., Metzidakis I.T., Petrakis P.V., Sergentani C.K., Doulis A.G. (2019). Phylogenetic and evolutionary applications of analyzing endocarp morphological characters by classification binary tree and leaves by SSR markers for the characterization of olive germplasm. Tree Genet. Genomes.

[11] Avramidou E.V., Koubouris G.C., Petrakis P.V., Lambrou K.K., Metzidakis I.T., Doulis A.G. (2020). Classification Binary Trees with SSR Allelic Sizes: Combining Regression Trees with Genetic Molecular Data in Order to Characterize Genetic Diversity between Cultivars of *Olea europaea* L. Agronomy.

[12] Mahner M., Kary M. (1997). What exactly are genomes, genotypes and phenotypes?. And what about phenomes? J. Theor. Biol..

[13] Walter A., Finger R., Huber R., Buchmann N. (2017). Smart farming is key to developing sustainable agriculture. Proc. Natl. Acad. Sci. USA.

[14] Costa C., Schurr U., Loreto F., Menesatti P., Carpentier S. (2019). Plant phenotyping research trends, a science mapping approach. Front. Plant Sci..

[15] Pieruschka R., Schurr U. (2019). Plant phenotyping: Past, present and future. Plant Phenomics.

[16] Costa J.M., Marques da Silva J., Pinheiro C., Baron M., Mylona P., Centritto M., Haworth M., Loreto F., Uzilday B., Turkan I. (2019). Opportunities and Limitations of Crop Phenotyping in Southern European Countries. Front. Plant Sci..

[17] Koutsos T., Menexes G. (2019). Economic, agronomic, and environmental benefits from the adoption of precision agriculture technologies: A systematic review. Int. J. Agric. Environ. Inf. Syst..

[18] Koubouris G., Bouranis D., Vogiatzis E., Nejad A.R., Giday H., Tsaniklidis G., Ligoxigakis E.K., Blazakis K., Kalaitzis P., Fanourakis D. (2018). Leaf area estimation by considering leaf dimensions in olive tree. Sci. Hortic..

[19] Zapolska A., Kalaitzidis C., Markakis E., Ligoxigakis E., Koubouris G. (2020). Linear Discriminant Analysis of spectral measurements for discrimination between healthy and diseased trees of Olea europaea L. artificially infected by Fomitiporia mediterranea. Int. J. Remote Sens..

[20] Boshkovski B., Doupis G., Zapolska A., Kalaitzidis C., Koubouris G. (2022). Hyperspectral Imagery Detects Water Deficit and Salinity Effects on Photosynthesis and Antioxidant Enzyme Activity of Three Greek Olive Varieties. Sustainability.

[21] Li L., Zhang Q., Huang D. (2014). A review of imaging techniques for plant phenotyping. Sensors.

[22] Dadwal M., Banga V.K. Color image segmentation for fruit ripeness detection: A review. Proceedings of the 2nd International Conference on Electrical, Electronics and Civil Engineering.

[23] Blasco J., Aleixos N., Moltó E. (2007). Computer vision detection of peel defects in citrus by means of a region-oriented segmentation algorithm. J. Food Eng..

[24] Kang S.P., East A.R., Trujillo F.J. (2008). Colour vision system evaluation of bicolour fruit: A case study with “B74” mango. Postharvest Biol. Technol..

[25] Mendoza F., Aguilera J.M. (2004). Application of image analysis for classification of ripening bananas. Food Eng. Phys. Prop..

[26] Throop J.A., Aneshansley D.J., Anger W.C., Peterson D.L. (2005). Quality evaluation of apples based on surface defects: Development of an automated inspection system. Postharvest Biol. Technol..

[27] Liming X., Yanchao Z. (2010). Automated strawberry grading system based on image processing. Comput. Electron. Agric..

[28] Blasco J., Aleixos N., Moltó E. (2003). Machine vision system for automatic quality grading of fruit. Biosyst. Eng..

[29] Costa C., Menesatti P., Paglia G., Pallottino F., Aguzzi J., Rimatori V., Russo G., Recupero S., Reforgiato Recupero G. (2009). Quantitative evaluation of Tarocco sweet orange fruit shape using optoelectronic elliptic Fourier based analysis. Postharvest Biol. Technol..

[30] Abràmoff M.D., Magalhães P.J., Ram S.J. (2004). Image processing with ImageJ. Biophotonics Int..

[31] Vázquez-Arellano M., Griepentrog H.W., Reiser D., Paraforos D.S. (2016). 3-D imaging systems for agricultural applications—A review. Sensors.

[32] Godin C. (2000). Representing and encoding plant architecture: A review. Ann. For. Sci..

[33] Bernard A., Hamdy S., Le Corre L., Dirlewanger E., Lheureux F. (2020). 3D characterization of walnut morphological traits using X-ray computed tomography. Plant Methods.

[34] Rist F., Herzog K., Mack J., Richter R., Steinhage V., Töpfer R. (2018). High-precision phenotyping of grape bunch architecture using fast 3D sensor and automation. Sensors.

[35] Furbank R.T., Tester M. (2011). Phenomics—Technologies to relieve the phenotyping bottleneck. Trends Plant Sci..

[36] Sanz-Cortes F., Martinez-Calvo J., Badenes M.L., Bleiholder H., Hack H., Llacer G., Meier U. (2002). Phenological growth stages of olives trees (Olea europaea). Ann. App. Biol..

[37] He J.Q., Harrison R.J., Li B. (2017). A novel 3D imaging system for strawberry phenotyping. Plant Methods.

[38] Anders A., Choszcz D., Markowski P., Lipiński A.J., Kaliniewicz Z., Ślesicka E. (2019). Numerical modeling of the shape of agricultural products on the example of cucumber fruits. Sustainability.

[39] Dutagaci H., Rasti P., Galopin G., Rousseau D. (2020). ROSE-X: An annotated data set for evaluation of 3D plant organ segmentation methods. Plant Methods.

[40] Paulus S. (2019). Measuring crops in 3D: Using geometry for plant phenotyping. Plant Methods.

[41] Atefi A., Ge Y., Pitla S., Schnable J. (2021). Robotic Technologies for High-Throughput Plant Phenotyping: Contemporary Reviews and Future Perspectives. Front. Plant Sci..

