# Supplementary material for: Introducing Three-Dimensional Scanning for Phenotyping of Olive Fruits Based on an Extensive Germplasm Survey
Source: Plants (Basel). 2022 Jun 2;11(11):1501. doi: 10.3390/plants11111501 (PMC9182883; doi:10.3390/plants11111501)
Supplement: Supplementary file 1 [file plants-11-01501-s001.zip › plants-1684642-supplementary.pdf]

Table S1. Endocarp 3D morphological traits of 50 olive varieties. Ten endocarp samples were analyzed for each variety (n=10)

Cultivar	Mean values ± Standard Error						
	Volume (cm3)	Area (cm²)	Up-skin (cm²)	Down-skin (cm²)	Center of gravity (size) x (cm)	Center of gravity (size) y	Center of gravity (size) z
Adramitini	0.69 ± 0.016 r-u	4.15 ± 0.070 o-s	0.20 ± 0.009 g	0.24 ± 0.013 ab	9.32 ± 0.064 n-r	9.35 ± 0.078 k-p	15.97 ± 0.234 i-p
Aggouromanakolia	0.49 ± 0.021 i-n	3.34 ± 0.108 i-n	0.08 ± 0.028 a-f	0.07 ± 0.024 a	8.09 ± 0.112 d-o	8.19 ± 0.095 d-m	15.43 ± 0.397 h-o
Amfissis	0.68 ± 0.065 q-t	4.20 ± 0.252 p-s	0.17 ± 0.044 a-e	0.11 ± 0.028 ab	9.35 ± 0.128 n-r	9.42 ± 0.335 l-p	16.83 ± 0.499 i-q
Amigdalolia	0.65 ± 0.032 p-s	4.28 ± 0.154 q-s	0.00 ± 0.000 a	0.10 ± 0.030 a	7.10 ± 0.050 h-p	8.63 ± 0.227 g-o	19.86 ± 0.572 s-t
Arbequina	0.27 ± 0.008 b-d	2.12 ± 0.040 b-d	0.18 ± 0.006 e-g	0.15 ± 0.009 ab	7.10 ± 0.050 b-i	7.13 ± 0.089 b-h	10.23 ± 0.225 ab
Asprolia Alexandroupolis	0.56 ± 0.027 l-r	3.65 ± 0.116 k-r	0.13 ± 0.032 b-f	0.18 ± 0.017 ab	9.30 ± 0.135 n-r	8.97 ± 0.218 i-p	15.10 ± 0.267 h-n
Asprolia Lefkados	0.28 ± 0.008 b-e	2.35 ± 0.042 b-f	0.00 ± 0.000 a	0.06 ± 0.019 a	6.48 ± 0.102 bc	6.46 ± 0.108 b-d	14.29 ± 0.112 e-j
Chalkidikis	0.87 ± 0.043 v-w	5.27 ± 0.166 u	0.07 ± 0.027 a-f	0.12 ± 0.029	10.99 ± 0.348 s	11.23 ± 0.178 q	19.77 ± 0.378

				a			r-s
Chondrolia Chalkidikis	0.66 ± 0.035 p-s	4.30 ± 0.151 r-t	0.06 ± 0.020 a-f	0.12 ± 0.021 a	9.96 ± 0.262 p-s	10.14 ± 0.166 n-q	17.45 ± 0.365 o-r
Chrisolia	0.32 ± 0.008 b-h	2.09 ± 0.209 bc	0.21 ± 0.014 g	0.22 ± 0.011 ab	8.31 ± 0.106 a	8.31 ± 0.094 e-m	9.12 ± 0.156 a
Dafnelia	0.417 ± 0.020 e-l	2.97 ± 0.097 e-l	0.17 ± 0.056 e-g	0.24 ± 0.039 ab	9.28 ± 0.322 n-r	9.41 ± 0.555 l-p	11.80 ± 0.854 b-d
Dopia Zakinthou	0.298 ± 0.010 b-f	2.40 ± 0.054 b-g	0.00 ± 0.000 a	0.07 ± 0.020 a	6.45 ± 0.671 a-c	7.01 ± 0.178 b-g	13.37 ± 0.316 d-h
Frantoio	0.354 ± 0.012 g-m	2.71 ± 0.059 c-i	0.00 ± 0.000 a	0.07 ± 0.020 a	7.48 ± 0.103 b-k	7.19 ± 0.116 b-h	14.75 ± 0.295 f-m
Frantoio Rodou	0.449 ± 0.014 a	3.14 ± 0.074 h-m	0.04 ± 0.019 ab	0.07 ± 0.023 a	8.27 ± 0.166 a	8.05 ± 0.119 c-l	15.05 ± 0.309 h-n
Gaidourelia	1.243 ± 0.42 x	7.20 ± 0.158 v	0.04 ± 0.029 a-c	0.06 ± 0.026 a	10.47 ± 0.173 rs	10.32 ± 0.337 o-q	28.93 ± 0.609 v
Galatistas	0.422 ± 0.033 a	3.06 ± 0.162 e-l	0.00 ± 0.000 a	0.04 ± 0.018 a	7.51 ± 0.244 b-k	7.62 ± 0.175 b-k	15.89 ± 0.437 i-p
Kalamon	0.613 ± 0.046 n-r	3.57 ± 0.411 k-q	0.00 ± 0.000 a	0.02 ± 0.016 a	8.10 ± 0.237 e-o	17.10 ± 1.474 r	10.45 ± 1.709 a-c
Kalokerida	0.489 ± 0.029 i-n	3.39 ± 0.130 i-n	0.03 ± 0.024 ab	0.10 ± 0.023 a	8.62 ± 0.262 i-p	9.20 ± 0.735 j-p	14.99 ± 0.602 g-n

Karolia	0.813 ± 0.017 t-v	5.05 ± 0.071 u	0.00 ± 0.000 a	0.00 ± 0.000 a	8.77 ± 0.121 i-q	8.74 ± 0.107 g-p	23.31 ± 0.253 u
Karidolia	0.909 ± 0.044 v-w	5.17 ± 0.157 u	0.03 ± 0.017 ab	0.02 ± 0.018 a	9.64 ± 0.163 o-s	9.82 ± 0.235 m-q	21.03 ± 0.292 tu
Kolireiki Ilias	0.498 ± 0.011 i-o	3.55 ± 0.061 k-p	0.00 ± 0.000 a	0.00 ± 0.000 a	7.72 ± 0.096 b-m	7.70 ± 0.100 b-l	17.99 ± 0.232 p-s
Kolimbada	1.011 ± 0.023 w	5.41 ± 0.094 u	0.11 ± 0.034 a-g	0.26 ± 0.016 ab	10.49 ± 0.990 rs	11.48 ± 0.117 q	16.62 ± 0.286 k-q
Koroneiki	0.121 ± 0.008 a	1.28 ± 0.040 a	0.00 ± 0.000 a	0.00 ± 0.000 a	4.92 ± 0.080 a	4.52 ± 0.408 a	10.42 ± 0.204 a-c
Kothreiki	0.596 ± 0.031 n-r	3.73 ± 0.138 m-r	0.13 ± 0.024 b-g	0.10 ± 0.020 a	8.87 ± 0.193 i-q	8.81 ± 0.168 h-p	15.21 ± 0.340 h-o
Koutsourelia	0.319 ± 0.014 b-h	2.30 ± 0.211 b-e	0.00 ± 0.000 a	0.00 ± 0.000 a	6.57 ± 0.634 b-e	7.11 ± 0.115 b-h	14.80 ± 0.309 f-m
Lefkolia Serron	0.49 ± 0.027 i-n	3.48 ± 0.119 j-o	0.00 ± 0.000 a	0.01 ± 0.012 a	6.96 ± 0.740 b-h	7.72 ± 0.150 b-l	18.66 ± 0.290 q-s
Lianolia Kerkiras	0.379 ± 0.021 c-j	2.96 ± 0.111 e-k	0.00 ± 0.000 a	0.00 ± 0.000 a	6.93 ± 0.193 b-h	6.66 ± 0.268 b-e	17.23 ± 0.418 n-q
Lianomanako Tirou	0.366 ± 0.008 c-e	2.75 ± 0.045 c-h	0.14 ± 0.006 b-g	0.11 ± 0.012 a	7.40 ± 0.073 b-j	7.47 ± 0.075 b-j	13.13 ± 0.150 d-h
Makris	0.40 ± 0.260 d-k	2.82 ± 0.118 d-j	0.17 ± 0.033 d-g	0.16 ± 0.023 ab	8.36 ± 0.215 g-o	8.44 ± 0.239	12.35 ± 0.185

						f-n	b-e
Manzanilla	0.517 ± 0.013 j-p	3.53 ± 0.056 j-p	0.15 ± 0.020 c-g	0.00 ± 0.000 a	8.17 ± 0.091 e-o	8.08 ± 0.112 c-m	16.92 ± 0.250 l-q
Mastoidis	0.284 ± 0.005 b-e	2.39 ± 0.033 b-g	0.00 ± 0.000 a	0.00 ± 0.000 a	6.29 ± 0.031 ab	6.27 ± 0.047 b	15.23 ± 0.182 h-o
Matolia Ilias	0.241 ± 0.010 a-c	2.07 ± 0.064 ab	0.00 ± 0.000 a	0.07 ± 0.019 a	6.39 ± 0.112 a-c	6.43 ± 0.106 bc	12.54 ± 0.175 b-f
Mavrelia	0.287 ± 0.014 b-f	2.39 ± 0.085 b-g	0.00 ± 0.000 a	0.02 ± 0.015 a	6.54 ± 0.120 b-d	6.51 ± 0.123 b-d	14.68 ± 0.298 f-l
Mavrelia Serron	0.557 ± 0.012 l-r	3.59 ± 0.047 k-r	0.10 ± 0.024 a-g	0.21 ± 0.011 ab	8.72 ± 0.118 i-q	8.79 ± 0.105 h-p	15.08 ± 0.219 h-n
Mavrelia Lefkadas	0.305 ± 0.013 b-g	2.43 ± 0.070 b-h	0.01 ± 0.011 a	0.03 ± 0.019 a	6.85 ± 0.097 b-g	6.79 ± 0.075 b-f	13.78 ± 0.231 d-i
Megaritiki	0.422 ± 0.009 e-l	3.21 ± 0.048 i-m	0.00 ± 0.000 a	0.02 ± 0.049 ab	7.36 ± 0.082 b-j	7.33 ± 0.063 b-i	18.21 ± 0.224 p-s
Mirtolia	0.451 ± 0.024 h-m	3.08 ± 0.110 g-m	0.10 ± 0.023 a-g	0.20 ± 0.031 ab	7.88 ± 0.173 c-n	7.87 ± 0.156 b-l	14.33 ± 0.229 e-k
Petrolia	0.88 ± 0.053 v-w	4.83 ± 0.190 s-u	0.36 ± 0.032 h	0.21 ± 0.031 ab	10.22 ± 0.256 p-s	10.43 ± 0.221 pq	16.42 ± 0.372 j-q
Picual	0.586 ± 0.023 m-r	3.99 ± 0.111 n-r	0.19 ± 0.016 b-g	0.08 ± 0.027 a	8.96 ± 0.210 k-r	8.43 ± 0.131 f-n	17.20 ± 0.254 n-q

Pierias	0.637 ± 0.024 o-r	4.02 ± 0.106 n-r	0.04 ± 0.021 ab	0.14 ± 0.026 ab	8.84 ± 0.155 i-q	8.15 ± 0.560 c-m	17.80 ± 0.328 p-s
Pierias Skotiniotiki	0.822 ± 0.031 u-v	4.77 ± 0.153 s-u	0.07 ± 0.026 a-f	0.20 ± 0.021 ab	9.27 ± 0.124 m-r	9.31 ± 0.203 k-p	20.02 ± 0.298 s-t
Pikrolia	0.43 ± 0.009 f-l	3.08 ± 0.042 g-m	0.14 ± 0.008 b-g	0.02 ± 0.015 a	7.69 ± 0.109 b-l	7.62 ± 0.095 b-k	14.81 ± 0.165 f-m
Rachati	0.22 ± 0.010 ab	1.93 ± 0.061 ab	0.00 ± 0.000 a	0.00 ± 0.000 a	6.37 ± 0.090 a-c	6.26 ± 0.089 ab	12.70 ± 0.304 c-g
Stroggilolia	0.648 ± 0.029 p-s	4.00 ± 0.011 n-r	0.05 ± 0.029 a-d	0.18 ± 0.012 ab	9.07 ± 0.215 l-r	9.08 ± 0.238 j-p	17.02 ± 0.298 m-q
Thiaki	0.25 ± 0.008 a-c	2.13 ± 0.048 b-d	0.00 ± 0.000 a	0.04 ± 0.018 a	6.69 ± 0.066 b-f	6.64 ± 0.060 b-e	12.28 ± 0.221 b-e
Throumbolia Thassou	0.519 ± 0.021 j-p	3.61 ± 0.093 k-r	0.01 ± 0.011 a	0.06 ± 0.021 a	7.84 ± 0.143 b-n	7.70 ± 0.143 b-l	18.45 ± 0.255 q-s
Throubolia	0.541 ± 0.013 k-q	3.67 ± 0.067 k-r	0.00 ± 0.000 a	0.08 ± 0.021 a	8.24 ± 0.088 f-o	8.05 ± 0.052 c-l	18.11 ± 0.317 p-s
Tragolia	0.243 ± 0.008 a-c	2.05 ± 0.045 bc	0.08 ± 0.018 a-f	0.04 ± 0.018 a	6.42 ± 0.071 a-c	6.46 ± 0.101 b-d	11.68 ± 0.190 b-d
Valanolia	0.559 ± 0.015 l-r	3.68 ± 0.057 l-r	0.06 ± 0.023 a-d	0.22 ± 0.030 ab	8.82 ± 0.177 i-q	8.59 ± 0.196 g-o	15.89 ± 0.237 i-p
Vasilikada	0.787 ± 0.032 s-v	5.01 ± 0.134 t-u	0.03 ± 0.023 ab	0.04 ± 0.022	8.86 ± 0.176	8.79 ± 0.180	21.57 ± 0.286

a i-q h-p tu

Source [(a)]	df	Volume (cm^3)	Area (cm^2)	Up-skin (cm^2)	Down-skin (cm^2)	Center of gravity (size) x (cm)	Center of gravity (size) y (cm)	Center of gravity (size) z (cm)
		F value						
Cultivar	49	81.998***	79.336***	15.599***	1.536*	23.011***	37.397***	76.022***

[(a)] Values of F: *P< 0.05 **P< 0.01 ***P< 0.001
[(b)] Mean values for each measured parameter within factor, with the same letter are not significantly different (P<0.05) LSD test.

Table S2. Fruit 3D morphological traits of 50 olive varieties. Five fruit samples were analyzed for each variety (n=5).

Cultivars	Volume (cm^3)	Area (cm^2)	Up-skin (cm^2)	Down-skin (cm^2)	Center of gravity (size) x (cm)	Center of gravity (size) y (cm)	Center of gravity (size) z (cm)
	Mean values ± Standard Error						
Adramitini	3.21 ± 0.136 d-h	10.96 ± 0.336 h-k	0.95 ± 0.045 h-m	1.08 ± 0.039 a-k	16.95 ± 0.289 i-p	16.98 ± 0.342 k-n	21.24 ± 0.371 e-i
Aggouromanakolia	2.44 ± 0.124 b-g	9.22 ± 0.331 e-j	0.80 ± 0.029 f-l	0.76 ± 0.009 a-i	15.00 ± 0.204 d-l	15.07 ± 0.256 g-k	20.16 ± 0.626 c-g
Amfissis	8.91 ± 0.177 o	21.59 ± 0.277 s	2.43 ± 0.065 v	1.82 ± 0.079 j-l	23.49 ± 0.225 t-w	24.05 ± 0.262 u-w	29.49 ± 0.432 p-r
Amigdalolia	11.22 ± 0.505 p	25.95 ± 0.728 s	1.37± 0.147 n-q	1.62 ± 0.109 f-l	24.04 ± 0.459 u-w	24.23 ± 0.415 u-w	38.89 ± 0.529 u
Arbequina	2.00 ± 0.097 a-e	7.94 ± 0.258 b-g	0.96 ± 0.022 h-m	0.80 ± 0.024 a-g	14.72 ± 0.246 c-k	14.46 ± 0.186 e-j	16.98 ± 0.218 b

Asprolia Alexandroupolis	5.95 ± 0.236 j-m	16.39 ± 0.432 n-p	2.07 ± 0.084 t-v	1.80 ± 0.074 i-l	21.56 ± 0.540 q-v	22.04 ± 0.210 s-u	23.43 ± 0.369 h-l
Asprolia Lefkados	0.98 ± 0.066 ab	5.09 ± 0.252 ab	0.21 ± 0.028 a	0.30 ± 0.024 a	10.31 ± 0.178 ab	10.62 ± 0.295 ab	17.71 ± 0.436 b-d
Chalkidikis	8.15 ± 0.369 no	20.65 ± 0.707 rs	1.87 ± 0.131 r-u	1.49 ± 0.117 e-l	18.68 ± 3.958 l-r	22.52 ± 0.323 t-v	31.40 ± 0.407 rs
Chondrolia Chalkidikis	10.86 ± 0.930 p	24.83 ± 1.325 t	2.19 ± 0.102 uv	1.94 ± 0.172 k-l	24.52 ± 0.841 uw	24.29 ± 0.895 vw	33.90 ± 0.563 st
Chrisolia	0.48 ± 0.062 a	3.01 ± 0.264 a	0.42 ± 0.046 a-e	0.41 ± 0.047 a-d	9.56 ± 0.438 l	9.58 ± 0.451 a	9.93 ± 0.344 a
Dafnelia	1.30 ± 0.042 a-c	5.99 ± 0.132 b-d	0.51 ± 0.040 a-f	0.40 ± 0.030 a-d	12.14 ± 0.237 a-e	12.16 ± 0.130 b-d	17.00 ± 0.112 b
Dopia Zakinthou	1.19 ± 0.052 a-c	5.67 ± 0.152 a-c	0.37 ± 0.016 a-c	0.35 ± 0.036 ab	11.64 ± 0.251 a-d	11.58 ± 0.281 a-c	17.37 ± 0.167 b-d
Frantoio	1.75 ± 0.018 a-d	7.39 ± 0.050 b-g	1.75 ± 0.032 a-f	0.53 ± 0.019 a-e	13.09 ± 0.079 a-i	13.15 ± 0.103 c-g	19.47 ± 0.187 b-f
Frantoio Rodou	2.26 ± 0.067 b-f	8.81 ± 0.172 d-i	0.69 ± 0.053 c-i	1.75 ± 1.146 h-l	14.35 ± 0.281 c-k	14.03 ± 0.166 d-i	21.29 ± 0.350 e-i
Gaidourelia	11.28 ± 0.461 p	25.98 ± 0.701 s	1.11 ± 0.091 k-n	1.67 ± 0.087 g-l	23.58 ± 0.243 t-w	23.73 ± 0.311 u-w	39.99 ± 0.869 u
Galatistas	3.71 ± 0.343 f-i	12.15 ± 0.709 j-m	1.14 ± 0.042 l-n	0.86 ± 0.086 a-g	17.17 ± 0.517 j-p	17.14 ± 0.438 k-n	23.80 ± 0.921 i-n
Kalamon	3.80 ± 0.341 f-i	12.72 ± 0.705 k-m	0.99 ± 0.054 i-m	0.87 ± 0.220 a-j	17.15 ± 0.667 j-p	16.97 ± 0.966 k-n	26.17 ± 0.776 l-o
Kalokerida	3.77 ± 0.311 f-i	12.54 ± 0.700 k-m	0.98 ± 0.061 i-m	0.43 ± 0.178 a-d	16.93 ± 0.358 i-p	16.74 ± 0.428 km	26.80 ± 1.372 n-p
Karolia	6.95 ± 0.427 l-n	19.48 ± 0.863 q-s	1.41 ± 0.072 n-q	1.08 ± 0.063 a-k	20.45 ± 0.420 p-u	20.73 ± 0.519 q-t	31.82 ± 0.684 rs
Karidolia	12.06 ± 0.546 p	26.53 ± 0.827 s	1.96 ± 0.071 s-u	2.19 ± 0.137 l	25.62 ± 0.452 w	25.91 ± 0.368 w	35.00 ± 0.524 t
Kolireiki Ilias	4.55 ± 0.188 h-j	14.02 ± 0.387 l-n	1.30 ± 0.070 m-p	0.81 ± 0.021 a-g	18.08 ± 0.330 k-q	18.00 ± 0.140 l-o	26.24 ± 0.306 l-o
Kolimbada	6.29 ± 0.374 k-m	17.00 ± 0.664 o-q	1.80 ± 0.086 r-t	1.61 ± 0.159 f-l	21.05 ± 0.407 q-v	21.39 ± 0.603 r-t	25.95 ± 0.284 k-o

Koroneiki	1.42 ± 0.046 a-c	6.47 ± 0.133 b-e	0.46 ± 0.018 a-f	0.44 ± 0.021 a-d	12.37 ± 0.131 a-f	12.11 ± 0.085 b-d	19.06 ± 0.393 b-e
Kothreiki	5.62 ± 0.498 j-l	15.79 ± 0.907 n-p	1.84 ± 0.114 r-u	1.43 ± 0.108 d-l	20.46 ± 0.591 p-u	20.25 ± 0.778 p-s	24.81 ± 0.542 j-o
Koutsourelia	2.71 ± 0.065 c-g	10.13 ± 0.304 g-k	0.73 ± 0.033 c-j	0.62 ± 0.101 a-f	15.75 ± 0.245 e-m	15.39 ± 0.069 h-k	22.61 ± 0.292 f-j
Lefkolia Serron	8.09 ± 0.306 no	20.13 ± 0.474 rs	1.58 ± 0.143 o-r	1.75 ± 0.125 h-l	22.74 ± 0.381 k-l	22.48 ± 0.452 t-v	30.14 ± 0.531 qr
Lianolia Kerkiras	1.56 ± 0.122 a-c	6.95 ± 0.363 b-f	0.38 ± 0.029 a-d	0.33 ± 0.033 ab	12.29 ± 0.326 a-e	12.13 ± 0.379 b-d	20.46 ± 0.516 d-h
Lianomanako Tirou	2.05 ± 0.093 a-e	8.16 ± 0.241 c-i	0.77 ± 0.028 e-l	0.72 ± 0.037 a-k	14.54 ± 0.168 c-k	14.50 ± 0.315 f-j	18.19 ± 0.313 b-e
Makris	6.26 ± 0.190 k-m	16.97 ± 0.330 n-q	1.93 ± 0.073 r-u	1.84 ± 0.081 j-l	22.05 ± 0.425 r-w	22.07 ± 0.238 s-u	24.54 ± 0.028 j-n
Manzanilla	4.73 ± 0.273 h-k	14.18 ± 0.543 m-o	1.67 ± 0.065 q-s	1.20 ± 0.052 a-l	19.06 ± 0.316 m-s	19.15 ± 0.301 n-q	24.04 ± 0.549 i-n
Mastoidis	1.88 ± 0.033 a-d	7.84 ± 0.104 b-g	0.55 ± 0.011 a-f	0.43 ± 0.020 a-d	13.35 ± 0.109 a-j	13.25 ± 0.094 c-h	21.17 ± 0.305 e-i
Matolia Ilias	1.40 ± 0.160 a-c	6.31 ± 0.482 b-e	0.49 ± 0.017 a-f	0.50 ± 0.037 a-e	12.38 ± 0.387 a-f	12.28 ± 0.409 b-e	17.44 ± 0.852 b-d
Mavrelia	1.07 ± 0.043 ab	5.36 ± 0.140 a-c	0.31 ± 0.027 ab	0.36 ± 0.015 a-c	11.07 ± 0.107 a-c	10.95 ± 0.321 ab	17.13 ± 0.218 bc
Mavrelia Serron	5.02 ± 0.270 i-k	14.62 ± 0.534 m-o	1.64 ± 0.060 p-s	1.60 ± 0.038 f-l	20.28 ± 0.454 o-u	20.02 ± 0.202 o-s	23.22 ± 0.598 g-l
Mavrelia Lefkadas	1.51 ± 0.033 a-c	6.69 ± 0.091 b-f	0.57 ± 0.028 a-g	0.46 ± 0.039 a-e	12.83 ± 0.116 a-h	12.68 ± 0.159 b-f	17.94 ± 0.337 b-d
Megaritiki	3.54 ± 0.061 e-i	11.84 ± 0.149 j-m	0.62 ± 0.046 b-i	0.94 ± 0.024 a-k	16.74 ± 0.051 i-p	16.60 ± 0.132 j-m	24.90 ± 0.377 j-o
Mirtolia	1.95 ± 0.132 a-e	7.87 ± 0.371 b-g	0.60 ± 0.047 b-h	0.66 ± 0.033 a-g	14.07 ± 0.207 b-j	13.87 ± 0.335 d-h	18.84 ± 0.385 b-e
Petrolia	3.92 ± 0.148 g-i	12.58 ± 0.301 k-m	1.41 ± 0.063 n-q	1.09 ± 0.048 a-k	18.09 ± 0.477 k-q	17.96 ± 0.285 l-o	22.75 ± 0.318 g-j
Picual	3.23 ± 0.357 d-h	11.13 ± 0.807 i-l	0.99 ± 0.098 i-l	0.85 ± 0.060 a-g	16.40 ± 0.579 g-n	16.30 ± 0.458 j-m	22.95 ± 1.136 g-k

Pierias	5.70 ± 0.337 j-l	16.17 ± 0.646 n-p	1.25 ± 0.092 m-o	0.83 ± 0.204 a-g	19.83 ± 0.401 n-t	19.75 ± 0.590 o-r	27.82 ± 0.499 o-q
Pierias Skotiniotiki	4.73 ± 0.410 h-k	14.25 ± 0.840 m-o	1.08 ± 0.060 j-n	1.05 ± 0.064 a-k	18.13 ± 0.549 k-q	18.45 ± 0.360 m-p	26.62 ± 0.997 m-p
Pikrolia	2.00 ± 0.146 a-e	8.14 ± 0.404 c-h	0.62 ± 0.019 b-i	0.48 ± 0.010 a-e	13.74 ± 0.330 b-j	13.86 ± 0.239 d-h	20.17 ± 0.549 c-g
Rachati	2.50 ± 0.143 b-g	9.50 ± 0.366 f-j	0.57 ± 0.037 a-g	0.41 ± 0.058 a-d	14.70 ± 0.317 c-k	15.05 ± 0.319 g-k	23.56 ± 0.756 h-m
Stroggilolia	6.81± 0.184 l-n	18.16 ± 0.322 p-r	1.25 ± 0.060 m-o	1.37 ± 0.071 b-l	21.12 ± 0.229 q-v	21.06 ± 0.195 q-t	29.65 ± 0.478 p-r
Thiaki	1.26 ± 0.057 a-c	5.90 ± 0.186 a-d	0.49 ± 0.027 a-f	0.42 ± 0.037 a-d	12.66 ± 0.675 a-g	12.12 ± 0.177 b-d	16.76 ± 0.293 b
Throumbolia Thassou	3.57 ± 0.076 e-i	12.09 ± 0.187 j-m	0.71 ± 0.013 c-i	0.66 ± 0.061 a-g	16.57 ± 086 h-o	16.42 ± 0.175 j-m	26.69 ± 0.310 m-p
Throubolia	3.20 ± 0.065 d-h	11.06 ± 0.150 h-k	0.75 ± 0.042 d-k	0.91 ± 0.046 a-k	16.20 ± 0.166 f-n	16.20 ± 0.142 i-l	23.24 ± 0.305 g-l
Tragolia	1.41 ± 0.038 a-c	6.38 ± 0.127 b-e	0.51 ± 0.025 a-f	0.42 ± 0.016 a-d	12.28 ± 0.104 a-e	12.35 ± 0.106 b-f	17.74 ± 0.276 b-d
Valanolia	5.01 ± 0.471 i-k	14.76 ± 0.876 m-o	0.94 ± 0.099 g-m	1.33 ± 0.096 a-l	18.88 ± 0.569 m-s	19.14 ± 0.682 n-q	26.22 ± 0.707 l-o
Vasilikada	7.48 ± 0.254 m-o	19.48 ± 0.443 q-s	1.62 ± 0.025 p-s	1.41 ± 0.041 c-l	21.36 ± 0.201 q-v	21.07 ± 0.331 q-t	30.45 ± 0.409 qr

		F value						
Source [(a)]	df	Volume (cm^3)	Area (cm^2)	Up-skin (cm^2)	Down-skin (cm^2)	Center of gravity (size) x (cm)	Center of gravity (size) y (cm)	Center of gravity (size) z (cm)
Cultivars	49	113.398***	139.401***	76.752***	8.617***	37.958***	124.927***	121.106***

(a) Values of F: *P< 0.05 **P< 0.01 ***P< 0.001

(b) Mean values for each measured parameter within factor, with the same letter are not significantly different (P<0.05) LSD test.
